# Interplay of oxidative stress, cellular communication and signaling pathways in cancer

**DOI:** 10.1186/s12964-023-01398-5

**Published:** 2024-01-02

**Authors:** Muhammad Javed Iqbal, Ayesha Kabeer, Zaighum Abbas, Hamid Anees Siddiqui, Daniela Calina, Javad Sharifi-Rad, William C. Cho

**Affiliations:** 1 Department of Biotechnology, University of Sialkot, Sialkot, Punjab Pakistan; 2 Institute of Biochemistry and Biotechnology, University of Veterinary and Animal Sciences, Lahore, Pakistan; 3 Department of Clinical Pharmacy, University of Medicine and Pharmacy of Craiova, 200349 Craiova, Romania; 4 Facultad de Medicina, Universidad del Azuay, Cuenca, Ecuador; 5 Department of Clinical Oncology, Queen Elizabeth Hospital, Kowloon, Hong Kong

**Keywords:** Oxidative stress, Reactive oxygen species, Cancer, Carcinogenesis mechanisms

## Abstract

Cancer remains a significant global public health concern, with increasing incidence and mortality rates worldwide. Oxidative stress, characterized by the production of reactive oxygen species (ROS) within cells, plays a critical role in the development of cancer by affecting genomic stability and signaling pathways within the cellular microenvironment. Elevated levels of ROS disrupt cellular homeostasis and contribute to the loss of normal cellular functions, which are associated with the initiation and progression of various types of cancer. In this review, we have focused on elucidating the downstream signaling pathways that are influenced by oxidative stress and contribute to carcinogenesis. These pathways include p53, Keap1-NRF2, RB1, p21, APC, tumor suppressor genes, and cell type transitions. Dysregulation of these pathways can lead to uncontrolled cell growth, impaired DNA repair mechanisms, and evasion of cell death, all of which are hallmark features of cancer development. Therapeutic strategies aimed at targeting oxidative stress have emerged as a critical area of investigation for molecular biologists. The objective is to limit the response time of various types of cancer, including liver, breast, prostate, ovarian, and lung cancers. By modulating the redox balance and restoring cellular homeostasis, it may be possible to mitigate the damaging effects of oxidative stress and enhance the efficacy of cancer treatments. The development of targeted therapies and interventions that specifically address the impact of oxidative stress on cancer initiation and progression holds great promise in improving patient outcomes. These approaches may include antioxidant-based treatments, redox-modulating agents, and interventions that restore normal cellular function and signaling pathways affected by oxidative stress. In summary, understanding the role of oxidative stress in carcinogenesis and targeting this process through therapeutic interventions are of utmost importance in combating various types of cancer. Further research is needed to unravel the complex mechanisms underlying oxidative stress-related pathways and to develop effective strategies that can be translated into clinical applications for the management and treatment of cancer.

Video Abstract

**Supplementary Information:**

The online version contains supplementary material available at 10.1186/s12964-023-01398-5.

## Introduction

Carcinogenesis is a complex multistage process that incorporates genetic mutations and abnormal cell division [1]. The onset and progression of cancer are closely linked to the generation of oxidative stress within cells. Oxidative stress refers to an imbalance between the production of reactive oxygen species (ROS) and the antioxidant defense mechanisms in cells. Under normal physiological conditions, the production of ROS is balanced by the presence of antioxidants, which neutralize these reactive species and maintain cellular homeostasis [2]. However, various factors such as environmental toxins, radiation, inflammation, and metabolic processes can disrupt this balance and lead to an excessive production of ROS. ROS, including molecules such as superoxide radicals, hydrogen peroxide, and hydroxyl radicals, are highly reactive and can cause damage to cellular components such as DNA, proteins, and lipids [2]. This oxidative damage can lead to mutations in critical genes, alterations in signaling pathways, and impaired cellular functions. In the context of cancer, the accumulation of ROS-induced DNA damage can contribute to genetic instability and the development of malignant tumors [3]. Additionally, oxidative stress can promote cell proliferation, angiogenesis, and resistance to cell death, providing favorable conditions for tumor growth and metastasis. It is worth noting that while oxidative stress is associated with cancer development, it is also involved in various physiological processes, including immune responses and cell signaling [3]. The key factor lies in maintaining a delicate balance between the production of ROS and the antioxidant defense systems within cells [4]. Understanding the role of oxidative stress in cancer pathogenesis has spurred research efforts to develop antioxidant-based therapies and strategies to mitigate oxidative damage. These approaches aim to restore the redox balance within cells and enhance the efficacy of conventional cancer treatments [4].

In addition to Reactive Oxygen Species (ROS), Reactive Nitrogen Species (RNS) also play a significant role in oxidative stress [5]. RNS, such as nitric oxide (NO) and peroxynitrite (ONOO-), contribute to cellular damage and are involved in various signaling pathways related to cancer progression. Similar to ROS, RNS can modulate cell survival, induce DNA damage, and affect mitochondrial functions [6]. The interplay between ROS and RNS further complicates the oxidative stress landscape, highlighting the need for therapeutic strategies that target both species [5, 6].

**Fig. 1:**
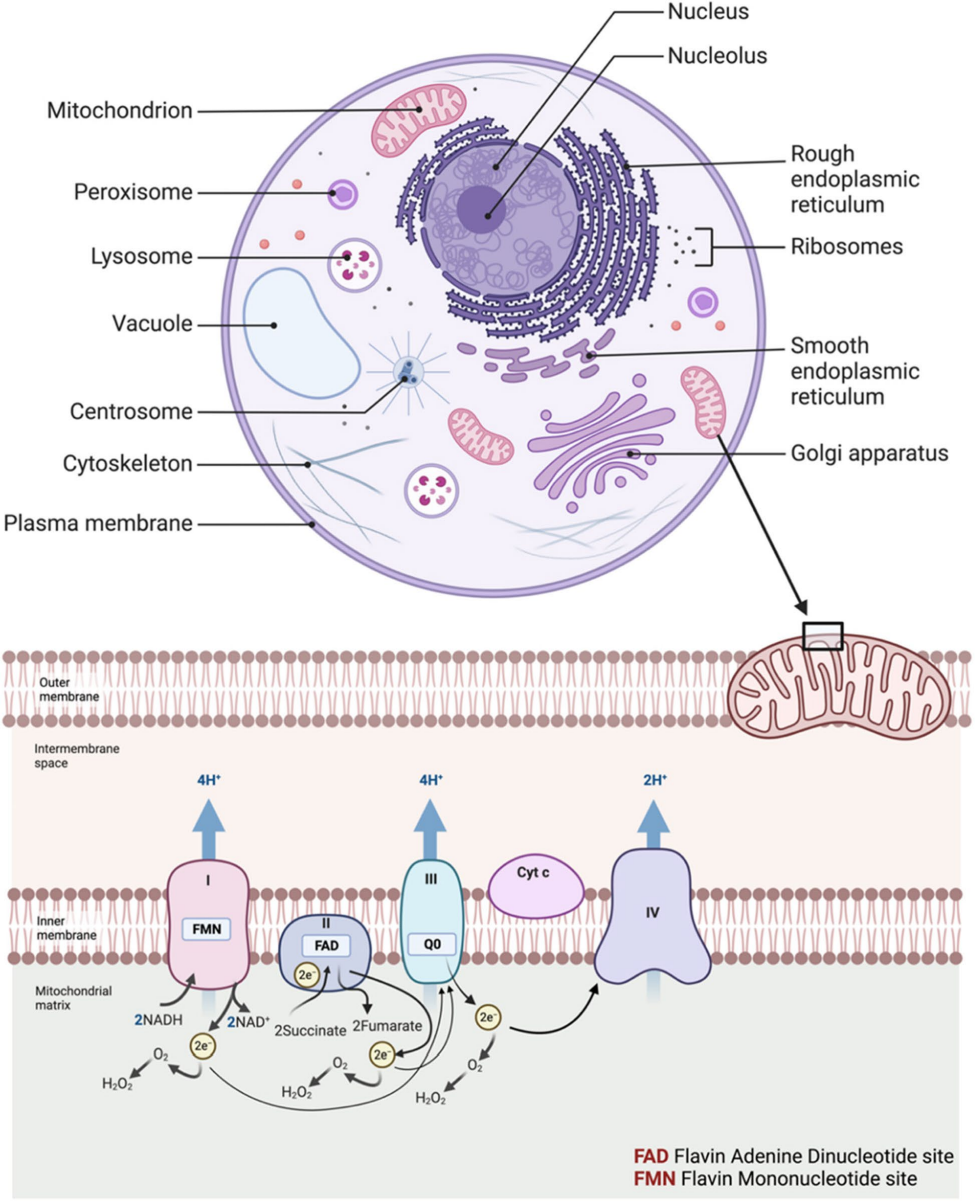
Production of ROS in cell. ROS is generated from different redox centers (FMN, FAD, Q_0_) in mitochondria

Oxidative stress acts as a potent catalyst in the transformation of normal cells into cancerous phenotypes, primarily by compromising genomic integrity. Elevated levels of reactive oxygen species (ROS), a hallmark of oxidative stress, interact with cellular macromolecules like DNA, RNA, and proteins. Specifically, ROS can induce DNA mutations, strand breaks, and even chromosomal aberrations by interacting with the nitrogenous bases and the sugar-phosphate backbone. Such genetic alterations disrupt the normal regulation of cell cycle, apoptosis, and DNA repair mechanisms. The compromised genomic integrity leads to the activation of oncogenes and the inactivation of tumor suppressor genes, thereby fostering an environment conducive for uncontrolled cell proliferation and tumorigenesis [7–10]. It is observed in various studies that oxidative stress is involved in the initiation and progression of various cancers, including melanoma, leukemia, lymphoma, oral, pancreatic, ovarian, bladder, breast, cervical, brain, gastric, liver, lung, and prostate cancer [11–14]*.* Reactive oxygen species (ROS), greatly produced in the mitochondria of cells (Fig. 1) [14], are observed as the main potential contributors to oxidative stress and cancer [15]. ROS are intracellular signaling molecules that contribute significantly to various signaling pathways, including insulin-like growth factor signaling pathway, and transient receptor potential channel-mediated cation signaling pathway [16]. In the growth factor signaling pathway, ROS, mainly H_2_O_2_ induces cell proliferation by inactivating protein and lipid phosphatases (i.e., PTP1B, PTPN2, PTPN11, PTEN) through the oxidation of cysteine residues present in their active site [17]. Whereas, in TRP channel-mediated cation signaling pathway, ROS accelerate Ca^2+^ signaling by activating a chain of enzymes belonging to the TRP protein family. Thus, inducing inflammation, proliferation, and cytoprotection that is followed by cell death [18, 19].

This review aims to discuss signaling pathways that are activated under high cellular oxidative stress and cause tumor progression. In addition to this, the regulation of ROS by tumor suppressor genes is discussed to highlight few significant treatment strategies to target oxidative stress in cancer.

### Oxidative stress, elevated ROS level and carcinogenesis: connecting the dots

ROS, such as hydrogen peroxide (H2O2), superoxide anion (O2-), and hydroxyl radical (OH*), are reactive oxygen-containing molecules that are generated as byproducts during cellular metabolism [20]. While ROS play essential roles in various cellular processes, excessive levels can have detrimental effects on cells and contribute to carcinogenesis. High concentrations of ROS can cause damage to cellular components, including DNA, proteins, and lipids [21]. This oxidative damage can lead to mutations in critical genes, including tumor suppressor genes, which normally help regulate cell growth and prevent the development of cancer. When the function of tumor suppressor genes is suppressed by ROS, it can disrupt the normal control mechanisms that prevent uncontrolled cell growth and proliferation. In addition to affecting tumor suppressor genes, ROS can also accelerate oncogenic signaling pathways [3]. Oncogenes are genes that, when mutated or overexpressed, can promote cell proliferation and survival, contributing to the development of cancer. ROS can activate these oncogenic signaling pathways, leading to enhanced cell growth, survival, and tumor formation [3]. The detrimental effects of ROS on cellular components and signaling pathways can create an environment conducive to carcinogenesis. Accumulation of DNA damage and genetic mutations, dysregulation of cellular signaling, and impaired antioxidant defense systems contribute to the initiation and progression of cancer [21]. It is important to note that ROS can also have physiological roles in cellular processes and signaling pathways under normal conditions. The effects of ROS on cell behavior are tightly regulated through a delicate balance between ROS production and the antioxidant defense system. However, when ROS levels exceed the cellular antioxidant capacity, oxidative stress occurs, leading to the disruption of cellular functions and contributing to carcinogenesis [22]. Understanding the role of ROS in cancer development is essential for developing strategies to prevent and treat cancer. Targeting ROS and oxidative stress pathways has been explored as a potential approach for cancer therapy [23]. Various antioxidant molecules and compounds that can scavenge ROS or modulate their levels are being investigated for their potential in inhibiting tumor growth and improving cancer treatment outcomes [24]. Thereby, creating oxidative stress and contribute to abnormal cell division and metastasis [25, 26]. Various biological processes, including epithelial-mesenchymal transition and angiogenesis in human body are seen to divert normal cell into cancerous cell due to exceeding cellular oxidative stress [27].

#### Epithelial-mesenchymal transition (EMT)

Epithelial-mesenchymal transition (EMT) event is associated with the conversion of epithelial cells into mesenchymal cells by acquiring motile and migratory characteristics. It is reported to be essential for the generation of body tissues during an individual’s development. Normally, epithelial cells are observed to retain apical-basal polarity and contact with adjoining cells through adherent junctions. Under oxidative stress, epithelial cells fail to maintain cellular polarity, cell-cell contact, undergone cytoskeletal modifications and initiate the transition into mesenchymal cells by acquiring migratory and invasive properties [28]. Various pathways like transforming growth factor beta (TGF-β) signaling, Wnt/β-catenin signaling, Notch signaling, and Hedgehog signaling are reported to stimulate EMT event [29]. Transcription factors, including Snail1, E12/E47, Zeb1/2, FOXC2, Slug, SIP1, Twist, Goosecoid, and epigenetic modifications like DNA methylation and remodeling of nucleosomes are also involved in the induction and initiation of EMT [29]. During the EMT process, mesenchymal markers that include fibronectin, N-cadherin, Snail, Slug, Twist, FOX C2, SOX 10, vimentin, MMP-2, MMP-3, and MMP-9 in epithelial cells are reported to be upregulated. Whereas, epithelial markers like E-cadherin, cytokeratin, desmoplakin, and occludin are reported to be downregulated, causing polarity loss, cytoskeletal reorganization, and generation of invasive phenotype in cells and facilitate the progression of cancer [28].

#### Angiogenesis

**Fig. 2:**
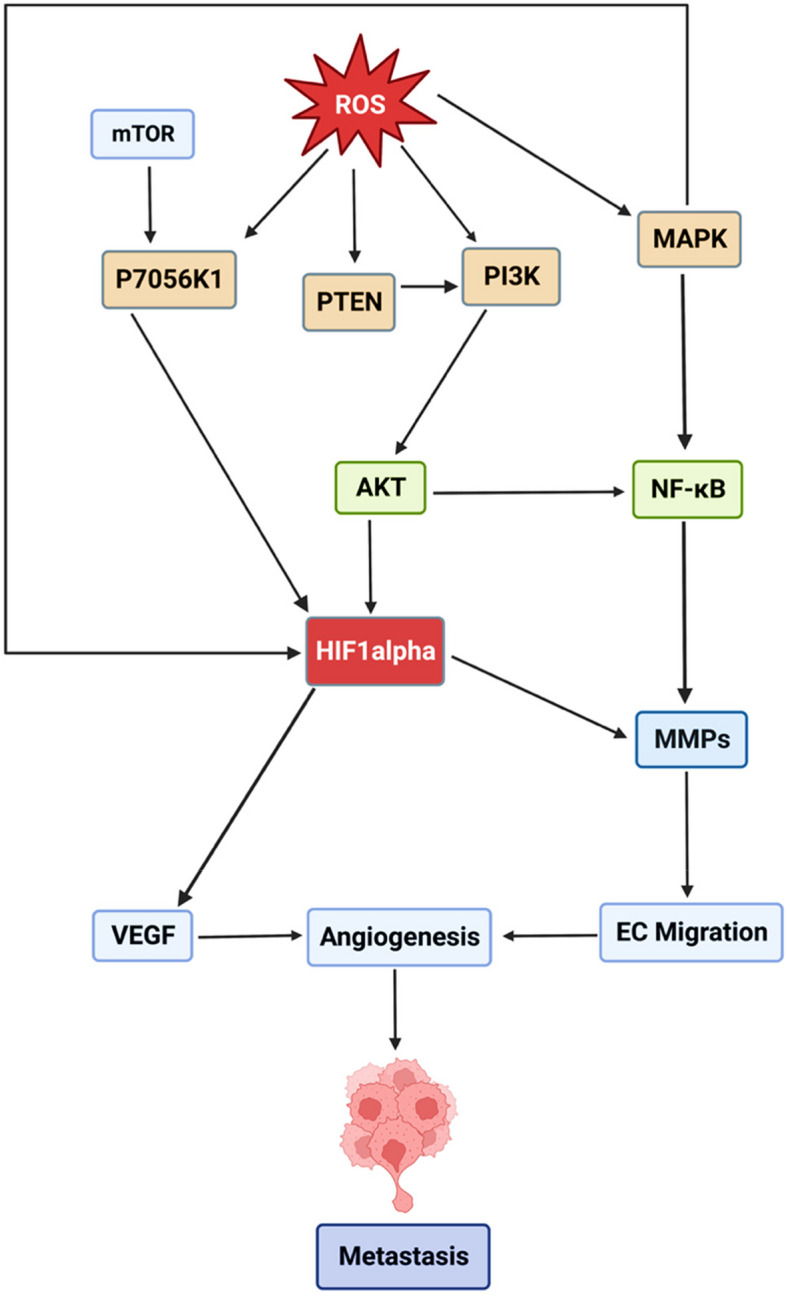
ROS and metastasis. ROS production stimulates the induction of PI3K/AKT/mTOR and MAPK pathways that trigger metastasis

Angiogenesis is the process by which new blood vessels are formed from pre-existing blood vessels. It is a complex biological process that plays a crucial role in various physiological and pathological conditions [30, 31]. In normal physiological processes, angiogenesis is essential for embryonic development, wound healing, and tissue repair. It occurs in response to specific signals and is tightly regulated to maintain tissue homeostasis. During angiogenesis, endothelial cells, which line the inner walls of blood vessels, undergo proliferation, migration, and remodeling to form new capillary sprouts [30]. In pathological conditions, such as cancer, angiogenesis becomes dysregulated and excessive. Tumor cells release various signaling molecules, including vascular endothelial growth factors (VEGFs), which stimulate angiogenesis and promote the formation of new blood vessels. The newly formed blood vessels supply the growing tumor with nutrients and oxygen, facilitating its growth and metastasis [32]. In tumorigenesis, the ROS dependent angiogenesis is initiated by the activation of PI3K/AKT/mTOR and MAPK pathways. In PI3K/AKT/mTOR pathway, phosphatidylinositol 3- kinases (PI3K) are reported to be activated in cells due to elevated cellular ROS level. These cascades lead to the activation of serine threonine kinases (AKT) which are reported to further activate hypoxia inducible factor 1 (HIF1α) and vascular endothelial growth factor (VEGF) to induce angiogenesis. On the other hand, mammalian target of rapamycin (mTOR) also triggers the activation of HIF1α and VEGF via ribosomal protein S6 kinase B1 (p70S6K1) under increased cellular oxidative stress. In MAPK signaling pathway, there is an activation of Mitogen-activated protein kinases (MAPK) due to increased cellular ROS level. Activated MAPK are reported to stimulate the production of nuclear factor kappa B (NF-κB) which triggers the release of cytokines and upregulation of matrix metalloproteinases (MMPs), causing angiogenesis and metastasis afterwards (Fig. 2) [33].

## Signaling pathways in oxidative stress and cancer

### MAPK pathway

Mitogen-activated protein kinases (MAPKs) are key signaling molecules in the cellular response to oxidative stress. ROS can activate MAPKs, leading to the phosphorylation and activation of downstream targets such as transcription factors AP-1 and NF-κB, which in turn regulate the expression of genes involved in cell survival, proliferation, and apoptosis﻿ [34].

### PI3K/AKT pathway

The phosphatidylinositol 3-kinase (PI3K)/AKT pathway is another crucial signaling axis affected by oxidative stress. ROS can directly activate PI3K, leading to the activation of AKT. The activated AKT can inhibit pro-apoptotic factors like Bad and caspase-9, promoting cell survival [35].

### Keap1-Nrf2 pathway

**Fig. 3:**
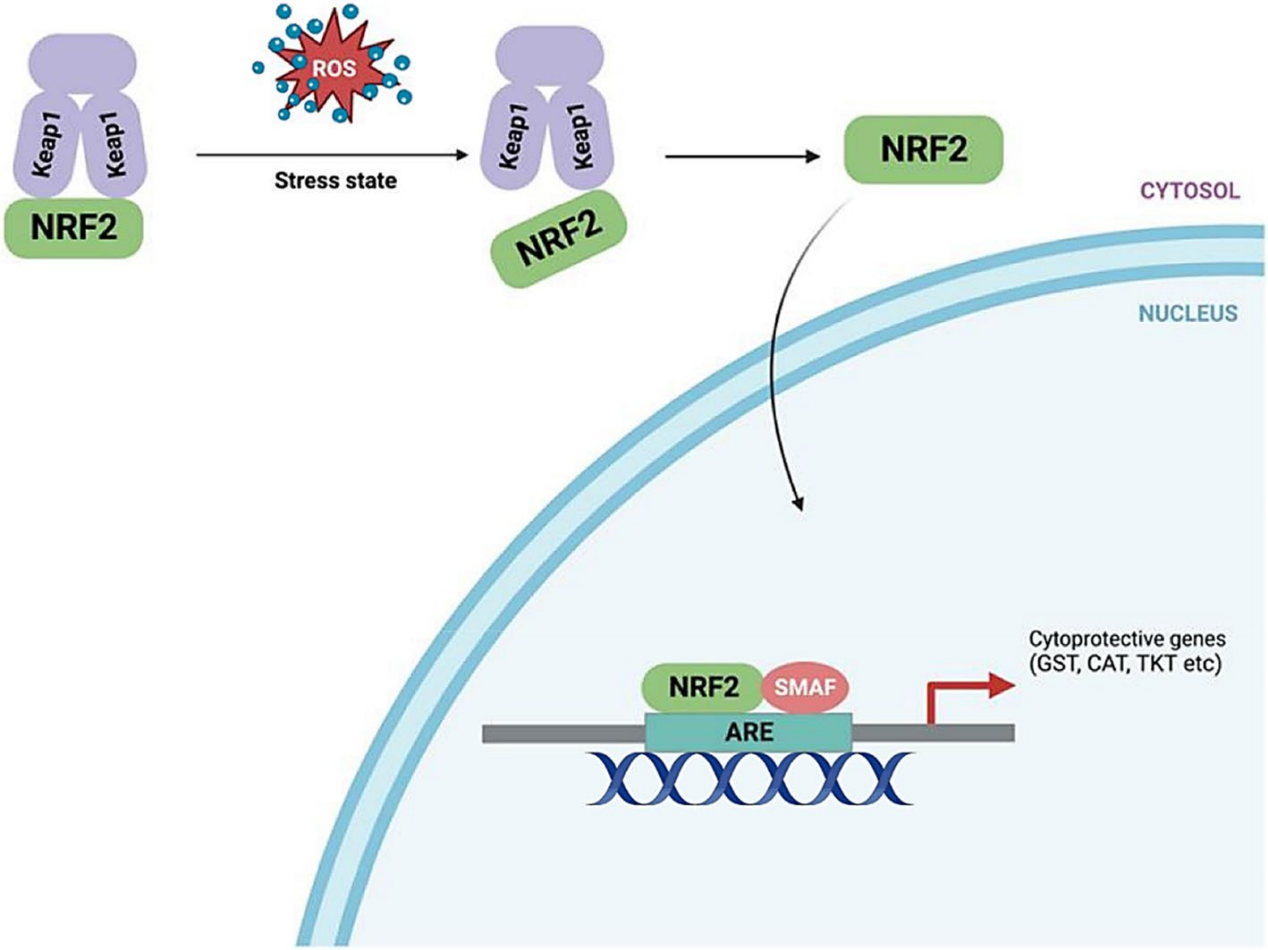
KEAP1-NRF2 pathway. Under oxidative stress condition, NRF2 detached from KEAP1 translocated to nucleus and trigger antioxidant response in cells by activating cytoprotective genes

Keap1-Nrf2 pathway is the main stress response pathway that is reported to be activated in cells in response to oxidative stress. It is comprised of four different and interlinked components that include chemical inducers (ROS), Kelch-like ECH-associated protein 1 (KEAP1), nuclear factor erythroid related factor 2 (NRF2), and target genes. Under normal cellular conditions, KEAP1 is reported to control the activity of NRF2 through NRF2 ubiquitination as well as proteasome-dependent degradation [36]. Whereas, under oxidative stress condition, NRF2 skips the ubiquitination process and translocate to the nucleus where it is reported to get attached to sMAF proteins and antioxidant response elements (ARE) to stimulate transcription program for the regulation of oxidative stress in cell (Fig. 3) [7, 36].

### JAK/STAT pathway

The Janus kinase (JAK)/Signal Transducer and Activator of Transcription (STAT) pathway is also susceptible to modulation by ROS [37]. Oxidative stress can induce the activation of JAKs, which in turn phosphorylate and activate STATs; activated STATs translocate to the nucleus and regulate the expression of genes involved in inflammation, cell proliferation, and apoptosis [37, 38].

### Wnt/β-catenin pathway

In the presence of oxidative stress, the Wnt/β-catenin pathway can be activated, leading to the accumulation and nuclear translocation of β-catenin; this promotes the transcription of target genes involved in cell proliferation and differentiation [39].

### p53 pathway

ROS can induce DNA damage, leading to the activation of the p53 pathway; activated p53 can either promote cell cycle arrest for DNA repair or induce apoptosis if the damage is irreparable [40].

Each of these pathways intricately interacts with oxidative stress, either amplifying its effects or mitigating its damage, and plays a significant role in the onset and progression of cancer [41].

## Tumor suppressor genes and oxidative stress: a mutual interplay in carcinogenesis

**Fig. 4:**
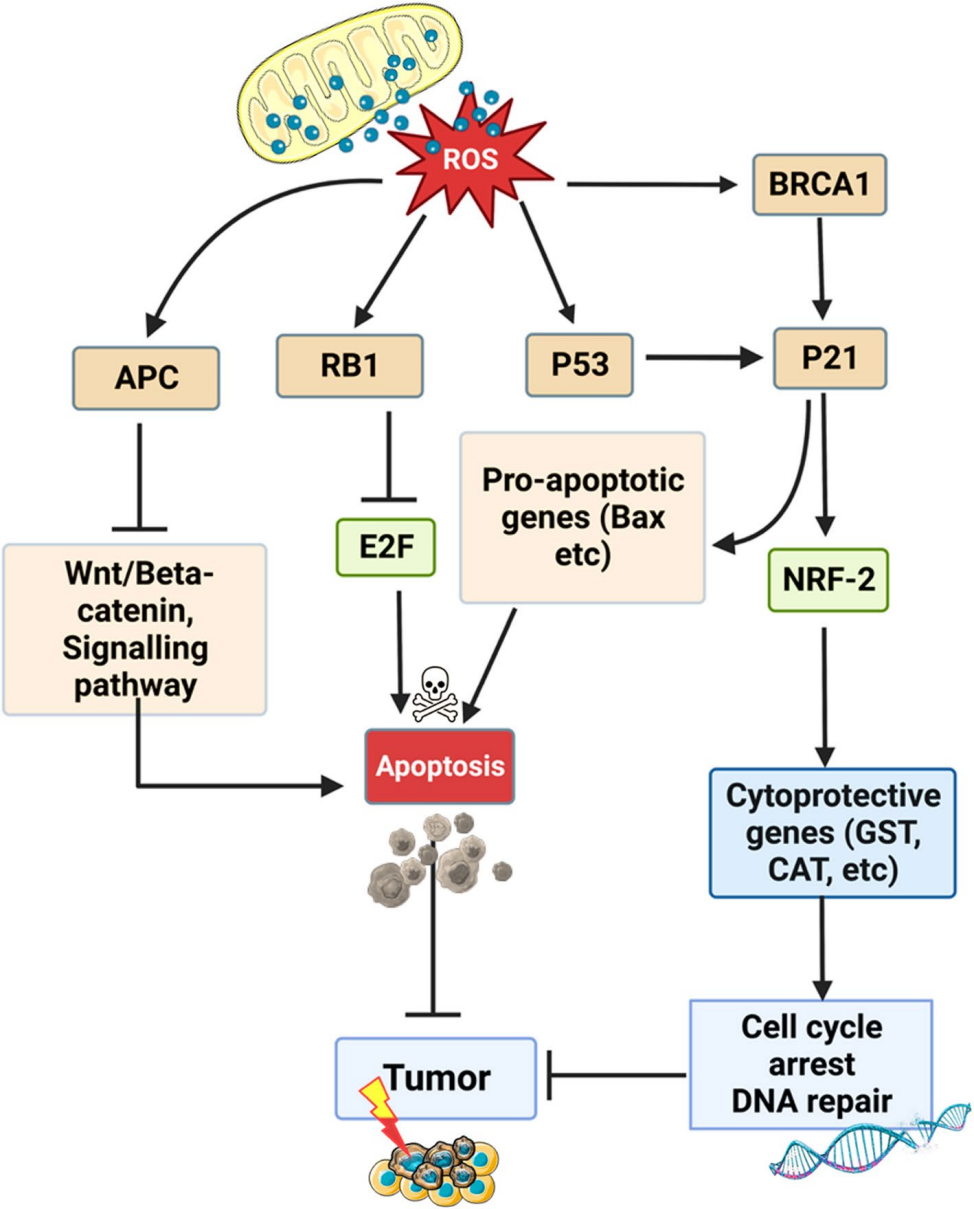
Regulation of ROS by tumor suppressor genes. In response to ROS, tumor suppressor genes activate the expression of antioxidant genes or prooxidative genes in cells for cell survival or apoptosis respectively, to prevent tumor growth

Cells employ a sophisticated array of mechanisms to counterbalance reactive oxygen species (ROS), oscillating between antioxidative strategies and the activation of tumor suppressor genes. These tumor suppressor genes serve not merely as passive barriers to tumorigenesis, but actively engage in the regulation of cellular processes; they control DNA repair mechanisms, enforce cell cycle checkpoints, and initiate apoptosis, thereby acting as cytoprotective agents. (Fig. 4) [42]. In the face of oxidative stress, tumor suppressor proteins act as pivotal regulators that dynamically modulate the cellular redox status [43]. These proteins can induce the transcription of antioxidant genes like glutathione peroxidases (GPx), superoxide dismutases (SOD), and catalases; simultaneously, they can suppress prooxidative genes that might otherwise exacerbate cellular stress and this dual regulatory ability enables tumor suppressor genes to create a finely tuned response that adapts to varying levels of oxidative stress [43, 44].

### p53

p53 is the chief regulator of programmed cell death and prevents tumorigenesis by facilitating the regulation of oxidative stress in cells. At low oxidative stress level, p53 promotes cell survival by stimulating the expression of antioxidant genes, including Parkin, sestrins 1/2, phosphate-activated glutaminase (GLS_2_), aldehyde dehydrogenase 4 (ALDH4), GPX1, and TIGER. At high oxidative stress levels, p53 promotes cell death by suppressing the expression of antioxidant genes and inducing the expression of prooxidative genes, including PIG3, PIG6, FDRX, Bax, Puma to further stimulate ROS production in cell that leads towards senescence [40, 45–47]. Inactivation of p53 is reported to be responsible for glioblastoma, retinoblastoma, neuroblastoma, medulloblastoma, lymphoma, bladder, pancreatic, breast, prostate, lungs, uterine, head and neck cancer [48–50].

### BRCA1 and BRCA2

Breast cancer susceptibility gene 1 (BRCA1) plays a significant role in the genomic stability of cells in response to oxidative DNA damage [51]. Under oxidative stress, the genomic integrity of the cell is compromised. BRCA1 is reported to regulate cellular oxidative stress by activating the expression of genes that encode paraoxonase 2 (PON2), Klotho (KL), ubiquitin carboxyl-terminal esterase L1 (UCHL1), glutathione S-transferase (GST), glutathione peroxidase (GPX3), alcohol dehydrogenase 5 (ADH5), and malic enzyme (ME2) [52–54]. Similar to BRCA1, BRCA2 is also reported to be involved in the regulation of cellular oxidative stress and protects DNA double-strand breaks [55]. Mutations in BRCA1 and BRCA2 genes are reported to be associated with breast, ovarian, esophageal, uterine, pancreatic, colorectal, cervical, stomach, prostate, and liver cancer [56].

### NRF2

The nuclear factor erythroid related factor 2 (NRF2) gene plays a critical role in tumor suppression by stimulating antioxidant response in cells against oxidative damage due to ROS [57]. It encodes NRF2 protein that is reported to initiate cytoprotective mechanism by binding with sMAF proteins and antioxidant response element (ARE) altogether in the nucleus. Thereby, activating the expression of cytoprotective genes encoding glutathione reductases, thioredoxin reductases, glutathione peroxidases, aldehyde dehydrogenases, transaldolases, transketolases, carbonyl reductases, ferritin light and heavy chains, thioredoxin, peroxiredoxin, sulfiredoxin, glutaredoxin, and malic enzymes to regulate oxidative stress [7, 58]. Pathogenic mutations in the NRF2 gene and their overexpression are reported to trigger colorectal, breast, liver, gall bladder, prostate, gastric, ovarian, and lung cancer [59, 60].

### RB1

Retinoblastoma transcriptional corepressor 1 (RB1) is a tumor suppressor gene that encodes RB1 protein. RB1 protein is reported to be responsible for maintaining cells’ genomic integrity in response to oxidative stress, thereby regulating the process of angiogenesis, apoptosis and cell cycle [61]. RB1 protein prevents tumorigenesis when dephosphorylated by protein phosphatase 2A (PP2A). Dephosphorylation allows RB1 protein to trigger cell quiescence by inhibiting the expression of E2f1, E2f2, and E2f3 transcription factors [62]. RB1 gene inactivation is reported to be involved in the induction and progression of retinoblastoma, glioblastoma, breast, prostate, lungs, and bladder cancer [61, 63].

### P21

The P21 gene, encoding the p21 protein is reported to help in tumor suppression by repairing DNA damage created due to oxidative stress. At low cellular ROS levels, p21 is reported to induce NRF2 dependent cytoprotective response to prevent cells from damage. At moderate ROS and oxidative stress levels, p21 triggers cell cycle arrest in between G1 and S phases to allow DNA to repair. However, at high cellular ROS level, p21 triggers the induction of pro-apoptotic response in cells by inhibiting NRF2-induced pro-survival response and causing cellular apoptosis [64]. It is noted that mutations in the p21 gene and its overexpression are responsible for causing gastric cancer, and esophageal squamous cell carcinoma [65, 66].

### APC

The adenomatous polyposis coli (APC) tumor suppressor gene is also reported to maintain the genomic stability of a cell by either DNA repair mechanism or mechanisms regulating cell death [67, 68]. At high cellular ROS concentration, activated expression of the APC gene is reported to hinder the base excision repair mechanism and Wnt/β-catenin signaling pathway. Thereby, facilitating apoptotic cell death and preventing cancer development and progression [67]. It is reported that mutations, including hypermethylation and deletions in the APC gene are responsible for triggering prostrate [69], gastric [70], pancreatic [71], and colorectal cancer [72–74]. Table 1 summarizes the roles and interactions of tumor suppressor genes with oxidative stress in various cancers.

**Table 1:** Interplay of tumor suppressor genes and oxidative stress: key regulators, functions, and associated cancers

Tumor Suppressor Gene	Low Oxidative Stress Action	High Oxidative Stress Action	Associated Cancers	References
**p53**	Promotes cell survival by stimulating antioxidant genes: Parkin, sestrins 1/2, GLS2, ALDH4, GPX1, TIGER	Promotes cell death by inducing prooxidative genes: PIG3, PIG6, FDRX, Bax, Puma	Glioblastoma, Retinoblastoma, Neuroblastoma, Medulloblastoma, Lymphoma, Bladder, Pancreatic, Breast, Prostate, Lungs, Uterine, Head and Neck	[47] [45]; [46] [40]; ;[48] [50]; ;[49];
**BRCA1/BRCA2**	Regulates oxidative stress by activating genes: PON2, KL, UCHL1, GST, GPX3, ADH5, ME2	Protects DNA from double-strand breaks	Breast, Ovarian, Esophageal, Uterine, Pancreatic, Colorectal, Cervical, Stomach, Prostate, and Liver	[51] [54]; [52]; [53] [55] [56];;;
**NRF2**	Initiates cytoprotective mechanisms by binding with sMAF proteins and ARE in the nucleus	Activates expression of cytoprotective genes	Colorectal, Breast, Liver, Gall Bladder, Prostate, Gastric, Ovarian, and Lung	[57] [7] [58] [60]; ;;[59];
**RB1**	Maintains genomic integrity in response to oxidative stress	Triggers cell quiescence by inhibiting expression of E2f1, E2f2, and E2f3	Retinoblastoma, Glioblastoma, Breast, Prostate, Lungs, and Bladder	[61] [62] [63]; ;[61];
**P21**	Induces NRF2-dependent cytoprotective response to prevent cell damage	Triggers induction of pro-apoptotic response	Gastric cancer, and Esophageal squamous cell carcinoma	[64] [65] [66];;
**APC**	Maintains genomic stability through DNA repair mechanisms	Hinders base excision repair mechanism and facilitates apoptotic cell death	Prostate, Gastric, Pancreatic, and Colorectal cancer	[68] [67]; [69]; [70]; [71] [72]; ;[74] [73];;

Legend: ↑ Increase or activation, ↓ Decrease or inhibition, *APC* Adenomatous Polyposis Coli, *ARE* Antioxidant Response Element, *ADH5* Alcohol Dehydrogenase 5, *ALDH4* Aldehyde Dehydrogenase 4, *BRCA1* Breast Cancer Susceptibility Gene 1, *BRCA2* Breast Cancer Susceptibility Gene 2, *E2f* E2 Factor, *FDRX* Ferritin Drug-Resistant X, *GLS2* Phosphate-Activated Glutaminase 2, *GPX* Glutathione Peroxidase, *GST* Glutathione S-Transferase, *KL* Klotho, *ME2* Malic Enzyme 2, *NRF2* Nuclear Factor Erythroid 2–Related Factor 2, *P21* Cyclin-Dependent Kinase Inhibitor 1, *PIG* p53-Inducible Gene, *PON2* Paraoxonase 2, *PP2A* Protein Phosphatase 2A, *RB1* Retinoblastoma 1, *ROS* Reactive Oxygen Species, *SOD* Superoxide Dismutase, *sMAF* Small MAF proteins, *TIGER* TP53 Induced Glycolysis Regulatory Phosphatase, *UCHL1* Ubiquitin C-Terminal Hydrolase L1

## Methods for oxidative stress profiling in oncology

Various approaches are reported to be utilized to evaluate the status of oxidative stress in clinical samples. Currently, the evaluation of oxidative stress in samples has been done in many ways, including direct measurement of ROS, assessment of oxidative damage, assessment of antioxidant status and other various methods.

In the direct measurement of ROS, fluorogenic probes (i.e., 5–6-carboxy-2,7-dichlorodihydrofluorescein diacetate and dihydroethidium) and d-ROMs test are reported to be used for the quantification of cellular ROS in a clinical sample; for the assessment of oxidative damage and antioxidant status, 2,4-dinitrophenylhydrazine (DNPH) assay and 2,2-diphenyl-1-picryl-hydrazyl (DPPH) reduction assay isreported to be used [75].

Molecular and biochemical assays such as 8-Oxo-2′-deoxyguanosine (8-Oxo-dG) measurements are used to quantify oxidative damage to DNA, a common feature in many cancer types [76]. Lipid Peroxidation Assays like Malondialdehyde (MDA) and 4-Hydroxynonenal (4-HNE) are used to assess oxidative damage to cellular lipids, which is implicated in cancer progression [77]. Genomic and transcriptomic approaches also offer valuable insights. RNA-Sequencing (RNA-Seq) identifies differentially expressed genes that are part of the oxidative stress response in cancer cells [78]. Clustered Regularly Interspaced Short Palindromic Repeats (CRISPR)/Cas9 screenings can identify genes that modulate sensitivity or resistance to oxidative stress, which is crucial for targeted therapy development [79].

Proteomic approaches like Redox Proteomics specifically identify proteins that undergo oxidative modifications, providing insights into cancer pathology [80]. Phosphoproteomics techniques identify oxidative stress-induced phosphorylation changes, crucial in oncogenic signaling pathways [81]. Metabolomic techniques such as targeted Liquid Chromatography-Tandem Mass Spectrometry (LC-MS/MS) are used for the quantification of specific metabolites like glutathione, directly involved in redox homeostasis [82]. Untargeted Metabolomics gives a comprehensive overview of metabolic changes due to oxidative stress and can provide potential biomarkers for cancer [83].

Imaging techniques like reactive oxygen species (ROS)-sensitive Magnetic Resonance Imaging (MRI) and Optical Imaging with ROS-sensitive probes are employed for in vivo visualization and real-time monitoring of ROS levels within tumors [84]. Cellular and tissue techniques like Immunohistochemistry (IHC) for oxidative stress markers and Cytofluorometric Analysis using fluorescent probes are used for quantifying intracellular levels of ROS or antioxidants [75]. In Silico and Computational Methods such as Pathway Analysis and Molecular Dynamics Simulations offer insights into ROS-induced signaling cascades and structural changes in biomolecules due to oxidative stress, respectively [85]. Liquid Biopsy approaches like circulating microRNAs (miRNAs) and cell-free DNA (cfDNA) can serve as non-invasive biomarkers for oxidative stress in cancer patients [86].

Table 2. summarizes the comprehensive methods currently employed for oxidative stress profiling in oncology, ranging from direct measurements of ROS to advanced genomic, transcriptomic, and metabolomic approaches.

**Table 2:** Comprehensive methods for oxidative stress profiling in oncology

Method Type	Description	Example Assays/Approaches	Reference
**Direct measurement of ROS**	Quantification of cellular ROS in clinical samples.	5–6-carboxy-2,7-dichlorodihydrofluorescein diacetate, dihydroethidium, d-ROMs test	[75].
**Oxidative Damage & Antioxidant Status**	Assessment of oxidative damage and antioxidant status.	2,4-dinitrophenylhydrazine (DNPH) assay, 2,2-diphenyl-1-picryl-hydrazyl (DPPH) reduction assay	
**Molecular & Biochemical Assays**	Quantification of oxidative damage to DNA.	8-Oxo-2′-deoxyguanosine (8-Oxo-dG)	[76]
**Lipid Peroxidation Assays**	Assessment of oxidative damage to cellular lipids.	Malondialdehyde (MDA), 4-Hydroxynonenal (4-HNE)	[77].
**Genomic & Transcriptomic**	Identification of differentially expressed genes related to oxidative stress.	RNA-Sequencing (RNA-Seq), CRISPR/Cas9 screenings	[78] [79]
**Proteomic Approaches**	Identification of proteins undergoing oxidative modifications.	Redox Proteomics	[80]
**Phosphoproteomics**	Identification of oxidative stress-induced phosphorylation changes.		[81]
**Metabolomic Techniques**	Quantification of specific metabolites like glutathione.	Targeted LC-MS/MS, Untargeted Metabolomics	[82] [83];
**Imaging Techniques**	In vivo visualization and real-time monitoring of ROS levels.	ROS-sensitive MRI, Optical Imaging with ROS-sensitive probes	[84]
**Cellular & Tissue Techniques**	Quantifying intracellular levels of ROS or antioxidants.	Immunohistochemistry (IHC), Cytofluorometric Analysis	[75]
**In Silico & Computational Methods**	Insights into ROS-induced signaling cascades and structural changes in biomolecules.	Pathway Analysis, Molecular Dynamics Simulations	[85]
**Liquid Biopsy**	Non-invasive biomarkers for oxidative stress.	Circulating microRNAs (miRNAs), cell-free DNA (cfDNA)	[86]

Abbreviations: *4-HNE* (4-Hydroxynonenal), *8-Oxo-dG* (8-Oxo-2′-deoxyguanosine), *cfDNA* (cell-free DNA), *CRISPR* (Clustered Regularly Interspaced Short Palindromic Repeats), *DNPH* (2,4-dinitrophenylhydrazine), *DPPH* (2,2-diphenyl-1-picryl-hydrazyl), *IHC* (Immunohistochemistry), *LC-MS/MS* (Liquid Chromatography-Tandem Mass Spectrometry), *MDA* (Malondialdehyde), *miRNAs* (microRNAs), *MRI* (Magnetic Resonance Imaging), *ROS* (Reactive Oxygen Species), *RNA-Seq* (RNA-Sequencing)

## Treatment approaches to target oxidative stress and cancer

Various treatment approaches have been incorporated to beat the cancer progression either in the form of chemotherapy, radiotherapy, hormonal therapy and combined therapies, to target various interlinked cancer signaling pathways. But still, there is a need for detailed molecular and machine learning approaches to introduce improved treatment strategies.

### ROS modulated therapeutic approaches

Recently, two ROS modulated therapeutic approaches are reported to be employed to target cellular oxidative stress for the prevention of various cancers. In ROS scavenging therapeutic approach, NADPH oxidase blocking agents, including diphenylene iodonium and apocynin, and various dietary antioxidants like polyphenols are reported to be incorporated to minimize the production and accumulation of cellular ROS. In ROS boosting therapeutic approach, increased concentration of nitroxide derivatives (i.e., nitroxide derived free radicals and cyclic nitroxides), or increased expression of glutathione S-transferases, superoxide dismutases (SODs), and catalases are reported to be utilized to target cellular oxidative stress and cancer [87–89].

ROS-modulated therapeutic strategies can be broadly classified into ROS-scavenging and ROS-boosting approaches, each with an array of agents acting through various mechanisms (Table 3).

i) ROS-Scavenging therapeutic -approaches [90, 91]

- NADPH oxidase inhibitors (e.g., Diphenylene iodonium, Apocynin) - inhibit the activity of NADPH oxidase, reducing the production of ROS.
- Antioxidant vitamins (e.g., Vitamin E, Vitamin C, Vitamin A) - neutralize free radicals by donating electrons, thereby reducing oxidative stress.
- Selenium compounds (e.g., Selenomethionine, Ebselen) - activate selenoproteins that function as antioxidants.
- Natural compounds (e.g., Quercetin, Resveratrol, Curcumin, EGCG) - these phytochemicals exert antioxidant effects by inhibiting ROS-generating enzymes and chelating metal ions.
- Enzyme mimetics (e.g., Manganese Porphyrins, EUK-134) - are synthetic compounds mimic natural antioxidant enzymes.
- Polyamines (e.g., Spermine, Spermidine) - modulate cellular redox status by chelating metal ions or inducing expression of antioxidant enzymes.
- Miscellaneous (e.g., Edaravone, Trolox, Tempol) - these agents work through various mechanisms, including free radical scavenging and metal chelation.

ii) ROS-Boosting therapeutic approaches [92, 93]

- Nitroxide derivatives (e.g., Tempol, Tempone) - generate ROS to induce oxidative stress in cancer cells.
- Pro-oxidant drugs (e.g., Arsenic trioxide, Doxorubicin, Menadione, Elesclomol) - create an imbalance in redox homeostasis, leading to elevated ROS levels and subsequent apoptosis.
- Photodynamic therapy agents (e.g., Aminolevulinic acid, Methylene blue, Rose Bengal) - produce ROS when activated by light, leading to oxidative damage.
- Natural pro-oxidants (e.g., Beta-Lapachone, Parthenolide, Capsaicin) - these natural compounds induce ROS generation, disrupting redox balance and leading to cell death.
- Melatonin: acts as a direct free radical scavenger and also stimulates antioxidant enzymes.
- Metal chelators (e.g., Deferoxamine, Triapine, L1) - chelate transition metal ions that catalyze ROS formation.
- Thiol antioxidants (e.g., N-Acetylcysteine, Glutathione, Thioredoxin) - donate electrons to neutralize free radicals.
- Redox-cycling drugs (e.g., Plumbagin, Juglone, Thiosemicarbazones) - these agents cycle between oxidized and reduced forms, generating ROS in the process.
- Increased expression or administration of antioxidant enzymes (e.g., SODs, Catalases, Glutathione S-Transferases, Peroxiredoxins) - these approaches involve the use of gene therapy or direct enzyme administration to elevate antioxidant enzyme levels, paradoxically generating ROS in cancer cells.
- Ionophores (e.g., Gramicidin, Valinomycin) - disrupt ion gradients across membranes, indirectly leading to ROS generation.
- Miscellaneous (e.g., Piperlongumine, PEITC, DATS) - have unique mechanisms, often involving modulation of redox-sensitive signaling pathways.\

**Table 3:** Overview of ROS-modulated therapeutic approaches for targeting oxidative stress in cancer

Therapeutic Approach	Agent Type	Examples	Mechanism	References
**ROS-Scavenging**	NADPH oxidase inhibitors	Diphenylene iodonium, Apocynin	inhibit NADPH oxidase, reducing ROS production	[90], [91]
	Antioxidant vitamins	Vitamin E, Vitamin C, Vitamin A	neutralize free radicals by donating electrons	
	Selenium compounds	Selenomethionine, Ebselen	activate selenoproteins functioning as antioxidants	
	Natural compounds	Quercetin, Resveratrol, Curcumin, EGCG	inhibit ROS-generating enzymes and chelate metal ions	
	Enzyme mimetics	Manganese Porphyrins, EUK-134	mimic natural antioxidant enzymes	
	Polyamines	Spermine, Spermidine	modulate cellular redox status by chelating metal ions or inducing expression of antioxidant enzymes	
	Miscellaneous	Edaravone, Trolox, Tempol	various mechanisms including free radical scavenging and metal chelation	
**ROS-Boosting**	Nitroxide derivatives	Tempol, Tempone	generate ros to induce oxidative stress	[92], [93]
	Pro-oxidant drugs	Arsenic trioxide, Doxorubicin, Menadione, Elesclomol	create redox imbalance, elevate ros levels leading to apoptosis	
	Photodynamic therapy agents	Aminolevulinic acid, Methylene blue, Rose Bengal	produce ROS when activated by light	
	Natural pro-oxidants	Beta-Lapachone, Parthenolide, Capsaicin	induce ROS generation disrupting redox balance	
	Metal chelators	Deferoxamine, Triapine, L1	chelate transition metal ions catalyzing ROS formation	
	Thiol antioxidants	N-Acetylcysteine, Glutathione, Thioredoxin	donate electrons to neutralize free radicals	
	Redox-cycling drugs	Plumbagin, Juglone, Thiosemicarbazones	cycle between oxidized and reduced forms, generating ros	

### Nanotechnology based treatment approaches

Nanotechnology based treatment strategy is the use of nanoparticles as a carrier for efficient therapeutic drug delivery on the destined spot; it is reported that nanocarrier based therapeutic dose delivery systems increase the therapeutic index of the drug with even small amount load, minimize system toxicity, and allow the drug to remain in the body for extended period to perform its therapeutic action towards cancer cells. Invitro experiments have shown that organic dye-doped silica NPs effectively target HepG2 liver cancer cells [94–96]. Besides, thermoresponsive chitosan-g-poly (N-vinylcaprolactam) NPs, and silver NPs are also reported to be utilized as anticancer drug carriers for efficient and effective delivery [97, 98]. Nanotechnology offers a sophisticated strategy for the targeted modulation of oxidative stress in cancer cells; utilizing various forms of nanocarriers, it is possible to either attenuate or exacerbate the cellular redox state, thereby influencing cancer cell fate [95].

i. ROS-scavenging nanocarriers

Cerium oxide nanoparticles: these nanoparticles act as regenerative antioxidants, mimicking the activity of both superoxide dismutase and catalase; once localized within the tumor microenvironment, they catalytically convert superoxide anions and hydrogen peroxide into harmless species, thus lowering intracellular ROS levels [99].

Manganese dioxide nanoparticles: these nanoparticles are activated in the acidic tumor microenvironment, where they catalyze the decomposition of hydrogen peroxide into oxygen and water, effectively reducing oxidative stress [100].

ii. ROS-generating nanocarriers

Gold Nanoparticles: upon irradiation with near-infrared light, gold nanoparticles generate heat that can induce the formation of ROS; the generated ROS can disrupt mitochondrial membranes, causing cytochrome c release and initiating apoptosis [101].

Copper Sulfide Nanoparticles: these nanoparticles, upon exposure to specific wavelengths of light, undergo electron-hole pair separation, leading to ROS generation, specifically singlet oxygen, which induces oxidative DNA damage and subsequent apoptosis [102].

iii. Dual-function nanocarriers

Polymeric nanocarriers with redox-responsive bonds: these nanocarriers encapsulate both ROS-generating and -scavenging agents. The disulfide bonds in the polymer matrix are cleaved in the high glutathione environment of cancer cells, releasing the encapsulated agents to modulate ROS levels dynamically [102, 103]

iv. Synergistic therapeutic strategies

Co-Delivery Systems: Nanocarriers such as liposomes can be engineered to encapsulate both chemotherapy agents like doxorubicin and antioxidant agents like curcumin. Doxorubicin induces ROS generation, while curcumin mitigates this effect in normal cells but enhances apoptosis in cancer cells through multiple pathways, including NF-κB inhibition [104].

### Advancing therapeutic interventions in oxidative stress and cancer

i. Targeted drug delivery systems: nanotechnology allows for the creation of nanoparticles like liposomes and polymeric micelles that can be functionalized with ligands such as antibodies or peptides [95]. These ligands have a high affinity for specific receptors overexpressed on cancer cells. Upon binding, these functionalized nanoparticles are internalized via receptor-mediated endocytosis, thereby ensuring the localized release of encapsulated ROS-modulating agents. This results in the targeted alteration of cellular redox balance, either by scavenging ROS with antioxidants or by generating ROS to induce cancer cell apoptosis [95].
ii. Epigenetic modulators: epigenetic drugs like 5-Azacitidine and Vorinostat act by inhibiting enzymes responsible for DNA methylation and histone deacetylation, respectively [105, 106]. These actions lead to the re-expression of genes that encode for antioxidants like glutathione and superoxide dismutases (SOD), thus altering the cellular redox state and making cancer cells more susceptible to oxidative stress-induced apoptosis [105].
iii. Enzyme inhibition strategies: specific inhibitors such as Allopurinol target xanthine oxidase, an enzyme involved in the conversion of hypoxanthine to xanthine and subsequently to uric acid, a process that generates ROS [107]. By inhibiting this enzyme, the cellular levels of ROS are reduced, which can inhibit the oxidative stress-induced signaling pathways that promote cancer cell proliferation [107].
iv. Immunotherapies: checkpoint inhibitors like anti-PD-1 antibodies function by blocking the interaction between PD-1 receptors on T cells and PD-L1 on cancer cells [108]. This blockage enhances the cytotoxic activity of T cells and produces cytokines that can induce oxidative stress in cancer cells, leading to apoptosis; this adds a new dimension to how immunotherapies can modulate the redox state within the tumor microenvironment [108].
v. Combination therapies: antioxidants such as N-Acetylcysteine (NAC) can mitigate the side effects of chemotherapy by donating electrons to free radicals generated by the drugs, neutralizing them [109, 110]. When used in conjunction with chemotherapy, this can both protect normal cells from oxidative damage and enhance the efficacy of the chemotherapy by allowing for higher tolerable doses [109, 110].

## Conclusions

Cancer continues to pose a substantial public health challenge, with diverse factors contributing to its onset and progression. One such pivotal factor is oxidative stress, mediated by the cellular production of reactive oxygen species (ROS). The role of ROS extends beyond being mere cellular cofactors and influences the onset of a wide array of cancers such as lymphoma, retinoblastoma, and various solid tumors including breast and lung cancer. They are implicated in key cellular processes such as epithelial-to-mesenchymal transition (EMT) and angiogenesis, which are precursors to metastasis. ROS modulation affects critical signaling pathways like Keap1-Nrf2, which traditionally regulates oxidative stress, and impacts tumor suppressor genes including p53, BRCA1, BRCA2, and RB1. These pathways and genes are either hyperactivated or inactivated under oxidative stress, leading to tumor growth and suppression, respectively. Current diagnostic approaches for oxidative stress, such as fluorogenic probes and d-ROMs tests, offer some insights but are not exhaustive. Likewise, existing treatment modalities like chemotherapy and radiotherapy have their limitations. Emerging strategies, such as ROS-modulated therapies and nanotechnology-based drug delivery systems, show promise in enhancing the effectiveness of anticancer drugs. Moreover, in accordance with the valuable suggestions received during the review process, we have expanded our discussion to include a wider range of ROS-modulatory agents, as reflected in a comprehensive table outlining these approaches. In summary, understanding the multifaceted role of oxidative stress in cancer biology is crucial for the development of more effective diagnostic tools and therapeutic interventions. Future research should focus on deciphering the complex interactions between oxidative stress and cellular pathways, with the aim of translating these findings into clinically applicable strategies for cancer management. This revised conclusion offers a more robust summary of the manuscript’s content, while laying out future directions for research in this area. Feel free to incorporate this into your manuscript.
